# Properties of Gaze Strategies Based on Eye–Head Coordination in a Ball-Catching Task

**DOI:** 10.3390/vision8020020

**Published:** 2024-04-15

**Authors:** Seiji Ono, Yusei Yoshimura, Ryosuke Shinkai, Tomohiro Kizuka

**Affiliations:** 1 Institute of Health and Sport Sciences, University of Tsukuba, Tsukuba 305-8574, Ibaraki, Japankizuka.tomohiro.ft@u.tsukuba.ac.jp (T.K.); 2 Graduate School of Comprehensive Human Sciences, University of Tsukuba, Tsukuba 305-8574, Ibaraki, Japan

**Keywords:** eye movements, head movements, gaze, ball-catching, motor skills

## Abstract

Visual motion information plays an important role in the control of movements in sports. Skilled ball players are thought to acquire accurate visual information by using an effective visual search strategy with eye and head movements. However, differences in catching ability and gaze movements due to sports experience and expertise have not been clarified. Therefore, the purpose of this study was to determine the characteristics of gaze strategies based on eye and head movements during a ball-catching task in athlete and novice groups. Participants were softball and tennis players and college students who were not experienced in ball sports (novice). They performed a one-handed catching task using a tennis ball-shooting machine, which was placed at 9 m in front of the participants, and two conditions were set depending on the height of the ball trajectory (high and low conditions). Their head and eye velocities were detected using a gyroscope and electrooculography (EOG) during the task. Our results showed that the upward head velocity and the downward eye velocity were lower in the softball group than in the tennis and novice groups. When the head was pitched upward, the downward eye velocity was induced from the vestibulo-ocular reflex (VOR) during ball catching. Therefore, it is suggested that skilled ball players have relatively stable head and eye movements, which may lead to an effective gaze strategy. An advantage of the stationary gaze in the softball group could be to acquire visual information about the surroundings other than the ball.

## 1. Introduction

In various sports situations, it is necessary to execute appropriate movements in response to visual targets such as a ball or opponent’s movements. Visual motion information plays an important role in the control of movements. Skilled ball players are thought to acquire accurate visual information by using an effective visual search strategy with eye and head movements. For example, when tracking a moving object, they do not track it all the way through, but rather capture a partial visual image, which is used to estimate the speed, trajectory, and spatial location of the ball. In previous studies on visual search, eye movements were mainly analyzed to evaluate the gaze, and the distribution of the gaze was clarified from the viewpoint of where the gaze was mostly placed in the visual field [1,2]. However, in actual sports situations, a visual search based on the interaction between eye and head movements is required. Especially in ball sports, it is necessary to react appropriately based on visual information, requiring not simply gazing at a visual target, but also acquiring other visual information while using appropriate visual strategies, including anticipatory skills [3].

Ball catching is a frequently used motor skill in ball sports such as baseball, basketball, and handball. In previous studies, most of the research on ball-catching skills has focused on motion analysis of catching. However, it has been suggested that the catching motion is closely related to the eye movement for tracking the ball [4,5,6]. Various studies have also been conducted on eye and gaze movements during ball tracking. Land et al. reported on gaze strategies during cricket batting, using an eye-movement-measuring device to record changes in eye position and the time spent gazing at the ball [7]. They revealed that batsmen need to predict the trajectory of the ball and decide the exact timing and position to hit the ball, and that more experienced players are more accurate in their responses. Lopez-Moliner et al. reported that the moment the ball is released is the moment when the ball’s trajectory can be predicted and the time when visual information is useful in catching [8]. In another study that examined how catchers and throwers looked at the ball when they had to both catch and find out what to do while the ball was approaching, they seemed to adjust their eye movements to the combined requirements of the task, rather than looking at the ball at a specific time [9].

Furthermore, since gaze behavior is determined using eye–head interactions, it is important to assess not only gaze behavior but also eye and head movements [10,11]. Thus, if gaze behavior differs across different skill levels, the eye and head movements associated with gaze behavior may also differ. This might reveal differences in detailed visual strategies that cannot be assessed using gaze behavior per se. During baseball batting, skilled players move their heads earlier and for longer in head translation toward the pitcher’s plate [12]. Regarding eye and head rotation, stable minimal eye motion is important for ball tracking in skilled baseball hitters even though head tracking motion becomes faster as the ball approached the batter [13]. The vestibulo-ocular reflex (VOR) is a reflexive eye movement induced by head rotation. In this case, eye movements are directed in the opposite direction of head movements and play an important role in maintaining the gaze on a stationary visual target [11,14]. However, the VOR is thought to interfere with the maintenance of the gaze position to a moving target. A recent study has reported that there was a significant negative correlation between the eye and head position, especially in the vertical direction, during table tennis rallies [15]. This result suggests that the horizontal VOR is suppressed more than the vertical VOR in ball tracking during table tennis forehand strokes. Other studies have shown that the VOR occurs during the first half of ball tracking, but the gaze position is firmly aligned with the ball [16]. This feature, called head tracking, effectively enables tracking by suppressing the VOR.

Several studies have reported the “quiet eye” as an effective eye movement in several sports, which is defined as the final fixation before the athlete initiates an execution at a specific location [17,18]. It has also been suggested that a short-term intervention method called “quiet eye training” is effective in improving performance [19]. Quiet eye training consists of viewing images of eye strategy instruction for throwing and catching tasks, and they reported an increase in the number of successful catches after viewing these images. In other words, subjects who had difficulty catching the ball showed greater improvement in catching the ball after learning gaze strategies. Therefore, it is possible that trained athletes and novices differ not only in their sports performance but also in their eye gaze strategies.

However, although there are differences in catching ability even in relatively simple behaviors, such as a ball-catching task, differences in catching ability and gaze movements due to sports experience have not been clarified. Therefore, by showing how eye and head movements are different from catching ability in different sports athletes, it would be possible to determine whether catching ability is due not only to differences in catching performance but also to differences in gaze behavior. Therefore, this study attempted to identify differences in catching skills and eye–head interactions among softball players who are accustomed to catching the ball, tennis players who are accustomed to tracking the ball but not catching the ball, and novice players who are neither familiar with catching the ball nor with tracking the ball. The purpose of this study was to determine the differences in gaze strategy among subjects with different ball-catching abilities. We focused on the eye and head interactions, including the VOR, during a simple catching task for different types of athletes and novices.

## 2. Methods

### 2.1. Participants

The participants were 19 female softball players who were accustomed to catching the ball (age: 23.3 ± 3.5 years; playing history: 14.2 ± 3.6 years; softball group), 15 female students of a university tennis club who habitually played tennis but no catching the ball (age: 20.7 ± 1.4 years; playing history: 10.2 ± 3.5 years; tennis group), and 15 female students who had not played a ball sport and had no regular exercise habits (age 21.7 ± 1.2 years; novice group). Thus, the total number of subjects was 49. All the subjects gave their informed consent to participate in the experiment. This study was conducted in accordance with the Declaration of Helsinki, and all protocols were approved by the Research Ethics Committee at the Institute of Health and Sport Sciences, University of Tsukuba.

### 2.2. Experimental Subject and Apparatus

**Figure 1:**
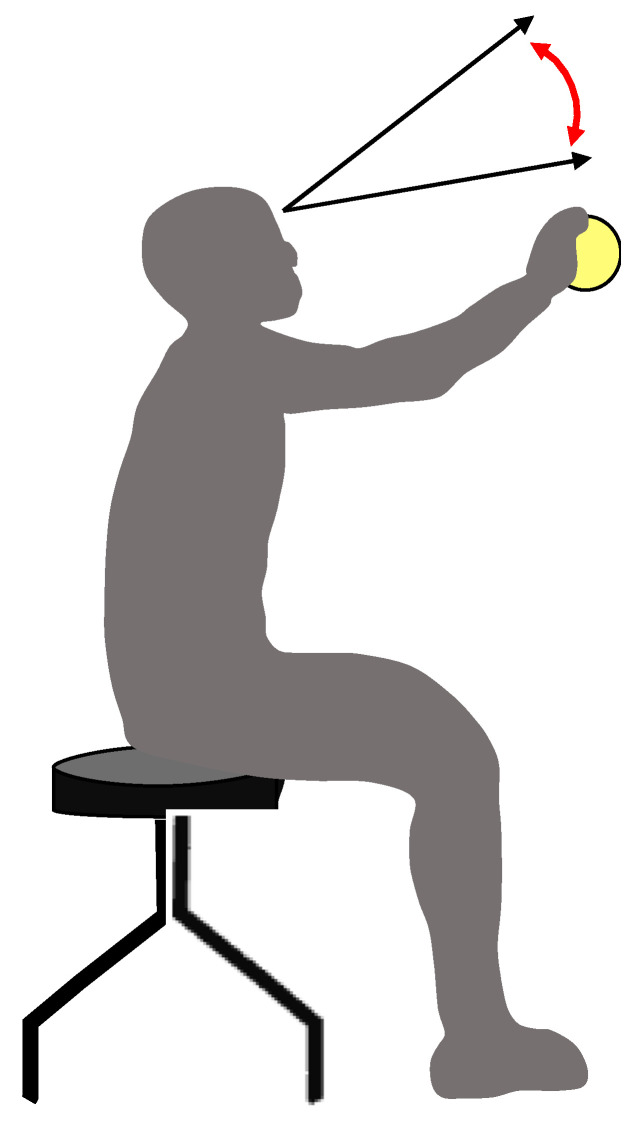
Measurement of the gaze–ball angle. Vertical head and eye movements were detected during a ball-catching task.

The experimental task was to catch a ball launched from the front (Figure 1). We attempted to detect eye and head velocity parameters during ball catching in order to assess eye and head coordination, including VOR. This is because VOR has been evaluated based on the eye and head velocity parameters, which usually deflect in opposite directions [11,14]. Therefore, subjects were fitted with a commercial wearable device, namely, smart eyeglasses with an integrated electro-oculography (EOG) and gyroscope (JINS MEME ES_R^®^, JINS Inc., Tokyo, Japan) [20]. Electrooculography was detected using the JINES MEME Data Logger software (ES_R) (JINS MEME ES_R^®^, JINS Inc., Tokyo, Japan). The device comprises electrodes and a voltage sensor for EOG recordings. The voltage is measured unipolarly with three electrodes located at the bridge (center, C), left (L) nose pad, and right (R) nose pad. The vertical (Vv) and horizontal (Vh) components can be obtained from the voltages continuously sampled at the three electrodes. The data mode was set to Full, the transmission rate to 100 Hz, and the measurement range from −1500 μV to +1500 μV. To detect the vertical eye movements, the component of time-series voltage waveforms of Vv was analyzed. Calibration was performed on a blackboard marked with 18 points (white circles with a diameter of 5 mm), and these points were 9 horizontal and vertical positions at 5° intervals in the viewing angle (0, 5, 10, 15, and 20°), respectively. Calibration was performed prior to the experiment, and subjects were asked to fixate on those points for at least 3 seconds, three times each during the EOG recording. To perform the calibration trials, the subjects sat 57 cm in front of the blackboard with the head stabilized using a chin rest and a forehead restraint. During the experiment (ball-catch task), head velocity was recorded using the built-in gyroscope, which detected angular velocity (frequency: 100 Hz) in the vertical axes in response to head rotation. Eye and head-recorded data were collected via wireless capabilities simultaneously and stored in a personal computer in the comma-separated value (CSV) file format. Eye and head velocity data were filtered using an 80-point finite impulse response (FIR) digital filter with a passband of 30 Hz in MATLAB R2016a (MathWorks, Natick, MA, USA). A tennis ball launcher (AP-LITE-DC, Sports Tutor Inc. Burbank CA, USA) was placed 9 m in front of the subject, and two high-speed video cameras (EX-ZR200, CASIO Inc. Tokyo, Japan) were placed behind and on the right of the subject to capture the test at 240 fps.

### 2.3. Experimental Conditions and Procedure

To examine the differences in head and eye movements depending on the height of the ball trajectory (upward visual angle), two conditions were set: a high trajectory (upward viewing angle: 31.1 deg; high condition) and a low trajectory (upward viewing angle: 21.0 deg; low condition). The upward viewing angle was calculated from the distance between the subject’s front-facing eye position and the highest point of arrival of the ball. The subjects sat in a chair adjusted so that their eye level was 125 cm from the floor in a sitting position. After 3 practice trials in each of the high and low conditions, 10 balls were caught in the main trial, for a total of 20 balls. The order in which each condition was performed was randomized.

### 2.4. Data Analysis

**Figure 2:**
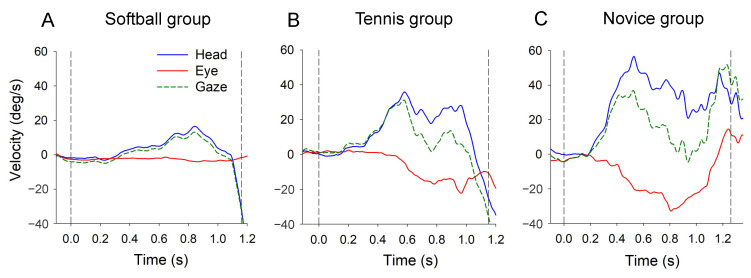
Typical examples of head velocity (blue), eye velocity (red), and gaze velocity (green) in the softball group (**A**), tennis group (**B**) and novice group (**C**) are shown. Two vertical dashed lines indicate the timing of the launch (left) and catch (right) of the ball.

To synchronize the timing of the launch and catch of the ball with the data, signals from infrared sensors were used. MATLAB R2016a was used to analyze the electrooculogram, and Upward Peak Head Velocity (UPHV) and downward Peak Eye Velocity (DPEV) were detected from each head and eye velocity trace between the time of the launch and catch of the ball (Figure 2). The catch rate was evaluated by dividing the number of successful attempts by the number of unsuccessful attempts. To compare differences between groups (soft, tennis, and novice groups) and conditions (high and low conditions), a two-way ANOVA and Bonferroni post-hoc test were performed on catch rate, UPHV, DPEV, and HA. Pearson product-moment correlation coefficients were calculated to examine the relationship between head and eye movements. All significance levels were less than 5%. SPSS Statistics 22 (IBM Inc., Armonk, NY, USA) was used for statistical processing.

## 3. Results

### 3.1. Comparison of Catch Rates between Groups

**Figure 3:**
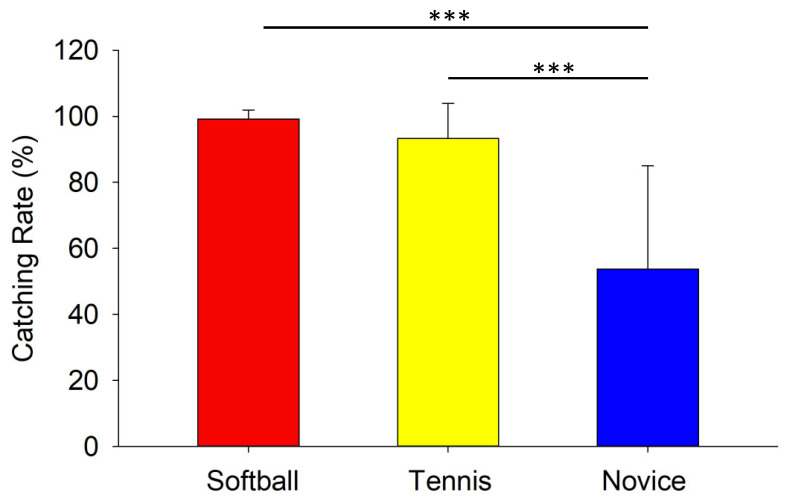
Comparison of the catching ratio of the softball, tennis, and novice groups for a ball-catching task. ***: *p* < 0.001.

The catch rate was compared using ANOVA, and a main effect was found for the groups (F (2,46) = 54.609, η2 = 0.48, *p* < 0.001). The results of the post-hoc test (99.21 ± 2.73% for softball, 93.33 ± 10.61% for tennis, and 53.75 ± 31.29% for novice) showed that the catch rate was significantly higher in the softball and tennis groups than in the novice group (*p* < 0.001) (Figure 3).

### 3.2. Comparison of Upward Peak Head Velocity (UPHV) between Groups and Conditions

To evaluate the head movements during the catch, an ANOVA comparing the UPHV showed a main effect for the groups (F (2,46) = 20.180, η2 = 0.43, *p* < 0.001). The results of the post-hoc test (21.69 ± 5.56 deg/s for softball, 31.12 ± 10.43 deg/s for tennis, and 38.82 ± 14.85 deg/s for novice) showed that the UPHV was significantly smaller in the softball group than the tennis and novice groups (*p* < 0.001), and the UPHV was significantly lower in the tennis group than the novice group (*p* = 0.003).

**Figure 4:**
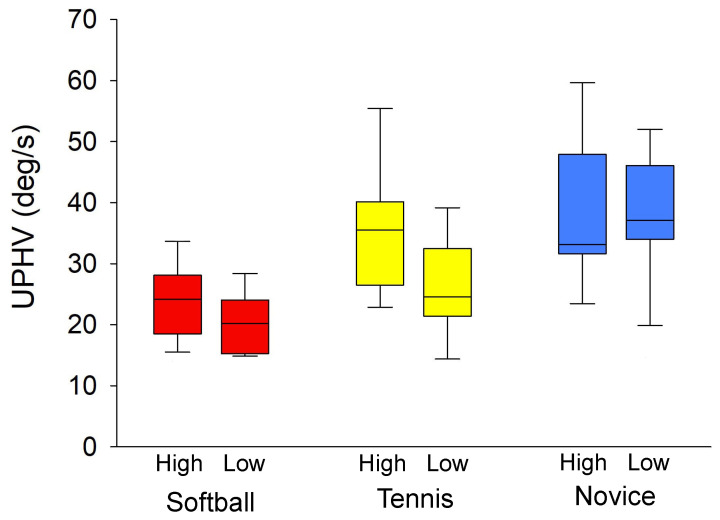
Comparison of the upward peak head velocity (UPHV) of the softball, tennis, and novice groups for a ball-catching task under high and low conditions. Box plots indicate 25–75 percentile ranges and central values, and error bars indicate 5–95 percentile ranges.

The UPHV was compared between conditions (softball, 23.28 ± 5.96 deg/s in the high condition, 20.10 ± 4.76 deg/s in the low condition; tennis, 35.98 ± 10.76 deg/s in the high condition, 26.26 ± 7.68 deg/s in the low condition; novice, 39.43 ± 12.20 deg/s in the high condition, 38.07 ± 17.48 deg/s in the low condition), indicating an interaction between group and condition (F (2,46) = 3.807, η2 = 0.05, *p* = 0.011). Furthermore, the results of the post-hoc test showed that the UPHV was significantly higher in the high condition than in the low condition in the tennis group (*p* < 0.001) (Figure 4).

### 3.3. Comparison of Downward Peak Eye Velocity (DPEV) between Groups and Conditions

The DPEV was compared using ANOVA and a main effect was found for the groups (F (2,30) = 10.630, η2 = 0.49, *p* < 0.001). The results of the post-hoc test (10.55 ± 3.78 deg/s for softball, 19.66 ± 6.83 deg/s for tennis, 24.37 ± 8.31 deg/s for novice) showed that the DPEV was significantly lower in the softball group than in the tennis and novice groups (*p* < 0.001).

**Figure 5:**
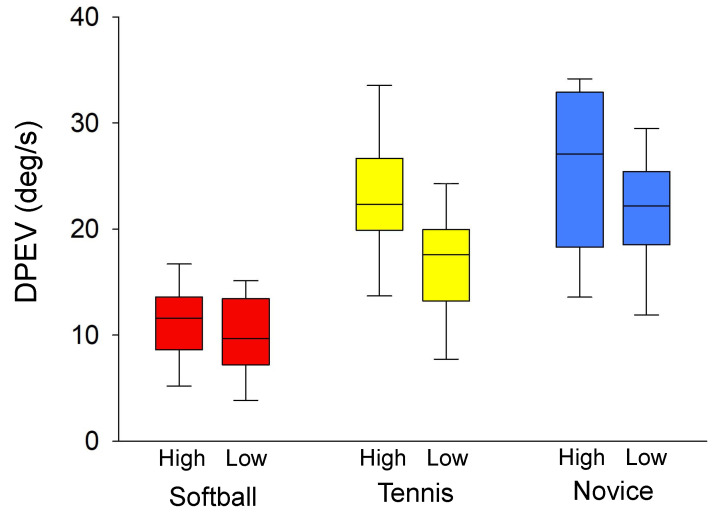
Comparison of the downward peak eye velocity (DPEV) of the softball, tennis, and novice groups for a ball-catching task under high and low conditions. Box plots indicate 25–75 percentile ranges and central values, and error bars indicate 5–95 percentile ranges.

The DPEV was compared between conditions (softball, 11.32 ± 3.82 deg/s in the high condition, 9.79 ± 3.66 deg/s in the low condition; tennis, 23.06 ± 6.43 deg/s in the high condition, 16.24 ± 5.51 deg/s in the low condition; novice, 24.75 ± 9.14 deg/s in the high condition, 24.06 ± 7.30 deg/s in the low condition), indicating an interaction between group and condition (*F* (2,30) = 3.481, η2 = 0.05, *p* = 0.007). Furthermore, the post-test results showed that DPEV was significantly larger in the high condition than in the low condition in the tennis group (*p* < 0.001) (Figure 5).

### 3.4. Relationship between Head and Eye Movements

**Figure 6:**
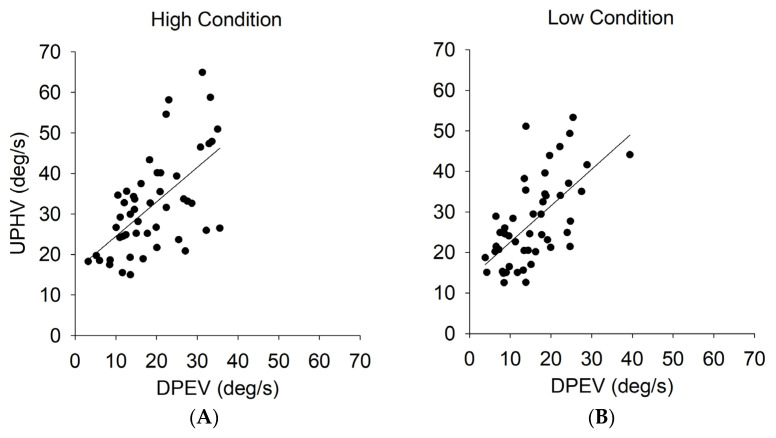
The relationship between the upward peak head velocity (UPHV) and the downward peak eye velocity (DPEV) under high (**A**) and low (**B**) conditions.

To examine the relationship between head and eye movements, we conducted a correlation between UPHV and DPEV for all participants. We found a significant positive correlation between the UPHV and the DPEV in the high condition (r = 0.381, *p* = 0.007) (Figure 6A). In addition, a significant positive correlation was found between the UPHV and the DPEV in the low condition (r = 0.520, *p* < 0.001) (Figure 6B). In addition, VOR gains based on the average UPHV and DPEV were lower for the softball group (0.48 in the high condition, 0.49 in the low condition) than for the tennis (0.64 in the high condition, 0.62 in the low condition) and novice (0.63 in the high condition, 0.63 in the low condition) groups.

### 3.5. Comparison of UPHV and DPEV between When Catching and Failing in Novices

The UPHV and the DPEV were compared in the novice group when they caught the ball and when they could not catch the ball. As a result, there was no difference in the UPHV between when catching (37.25 ± 9.45 deg/s) and failing (39.22 ± 10.68 deg/s) to catch the ball (*p* = 0.243). Similarly, there was no difference in the DPEV between when catching (21.92 ± 5.34 deg/s) and failing (24.12 ± 6.85 deg/s) to catch the ball (*p* = 0.143). 

## 4. Discussion

### 4.1. Catching Performance

In the catching task in this study, the participants performed a one-handed catch of a ball launched from the front. This type of action is expected to be performed in defensive situations in baseball and softball. When we compared the catch rates between the softball and tennis groups, we found no difference between these groups (Figure 3). One reason for this is that the task in this study was a relatively simple task in which the players caught the ball while seated. Nevertheless, the significant difference in the catch rates between the novice group and the other two groups may be due to the fact that the novice group had little experience with ball sports, which affected their basic catching ability. We then examined whether this difference in catching ability was related to differences in eye and head movements.

### 4.2. Head Movements in Catching the Ball

We measured the upward peak head velocity (UPHV) to evaluate head movements during the ball-catching task. Previous studies examining the main sequence of head movements demonstrated that there is a positive correlation between the peak velocity and the amplitude of movement [21]. Our results showed that the UPHV was significantly smaller for the softball group than for the tennis and novice groups, and it was also significantly smaller for the tennis group than for the novice group (Figure 3). This indicates that the head movements in the novice group showed a more upward pitch than the other groups. Since the novice group was not accustomed to observing the ball, they used head movements to move their gaze when catching the ball. Although there are previous studies that assessed eye and head movements in a vertical direction during the interceptive performance [7,15,22,23], this study quantitatively clarified significant differences in vertical head movements between different skill levels for ball catching. The smaller head movements in the softball players could be directly related to stationary gaze behavior, which may lead to not only stable visual information but also a stable catching motion/posture. A further notable result is that although softball and tennis players had similar catch rates, their head movements were markedly different. Therefore, the results suggest that head movements can be used to evaluate catching skills that are not reflected in the catch rate. For example, in a previous study that examined catching motion using a motion capture system, reaching and catching motions during ball catching were measured to evaluate catching ability [24]. It is possible that head movements are also linked to the catching motion.

Furthermore, the UPHV of the tennis group was significantly greater in the high condition than in the low condition (Figure 4). The tennis group showed higher head velocity when the trajectory was high and lower head velocity when the trajectory was low, suggesting that they adjusted their head movements according to the trajectory of the ball. Another possibility is that the tennis group is not accustomed to catching the ball in tennis games, so they tend to observe the ball with head movements, as the novices did. However, there was no difference in the UPHV of the softball and novice groups between the high and low conditions. The softball group showed relatively smaller values in both conditions, indicating that the head movement was small regardless of the height of the ball trajectory. In contrast, the novice group showed relatively larger values in both conditions, suggesting that the head moved more during the catch regardless of the height of the trajectory. 

### 4.3. Eye Movements in Catching the Ball

The DPEV was significantly larger in the novice group than in the tennis and softball groups. This result suggests that the head moved upward in response to the upward trajectory of the ball, which led to a downward vestibulo-oculomotor reflex (VOR). Furthermore, the DPEV was significantly larger in the high condition than in the low condition for the tennis group (Figure 5). In contrast, there was no difference in the DPEV for the softball and novice groups between the conditions. Furthermore, the softball group showed smaller values in both the high and low conditions, indicating that the eye movements were stable regardless of the ball trajectory. On the other hand, the novice group showed larger velocity in either condition, suggesting that downward eye movements were induced by the VOR regardless of the trajectory height. In the tennis group, the downward velocity was large when the trajectory was high and small when the trajectory was low, suggesting that the VOR was induced by larger head movements. Previous studies have reported that the function of the VOR is to minimize image stabilization on the retina by moving the eye in the opposite direction at the same speed when the head moves [25]. Such a mechanism plays a role in controlling retinal motion error signals by adjusting the gain and phase of the VOR to minimize blurring to accurately capture visual image [26]. However, while the VOR is an effective eye movement when viewing a stationary object, when viewing a moving object it is presumably more difficult to track the ball with eye movements moved in the opposite direction. Thus, the advantage of the small eye velocity and stationary gaze in the softball group is to acquire visual information about the surroundings other than the ball. In other words, it suggests that skilled players use a visual strategy that allows them to acquire other visual information, such as runners and defenders, during the catch by predicting the trajectory of the ball rather than tracking the entire ball trajectory. The possible reason for the smaller eye velocity in the softball group is that they utilized their peripheral vision and did not direct their gaze along the ball’s trajectory. This result is also consistent with a previous study that used baseball batters during fastball hitting [13]. In contrast, previous eye movement studies have reported that eye tracking a rapid change in velocity causes the eye to adapt to that velocity and that the eye velocity also increases with the movement of the eye [27,28]. However, the purpose of those experiments was to track the visual target, whereas the purpose of the current experiment was to catch the ball. Therefore, the eye movements were smaller in those who were accustomed to observing the ball while catching on a regular basis, suggesting that smaller head and eye movements lead to more effective visual strategies for a ball-catching task.

### 4.4. Relationship between Head and Eye Movements

To verify the relationship between head and eye movements associated with the VOR, the correlation between the UPHV and the DPEV was examined. The results showed a significant correlation between the UPHV and the DPEV in both high and low conditions (Figure 6). This result suggests that the faster the upward head movement, the faster the downward eye movement, that is, the faster the VOR in the direction opposite to the head movement. Thus, novice players, who are less accustomed to observing the ball, elicit a faster VOR, resulting in faster eye movements in the direction opposite to head movements. In contrast, the softball group is able to track the trajectory of the ball with their heads, suggesting that the VOR is suppressed, and they track the ball more efficiently as VOR suppression is an effective mechanism for tracking a moving target with head movements. In addition, our results showed that VOR gains based on the average UPHV and DPEV were lower for the softball group than for the tennis and novice groups. Previous studies have reported that VOR suppression occurs through inhibition of vestibular nerves in active head motion [11] or smooth pursuit signals in the opposite direction of the VOR [29]. The advantage of using such head tracking is that it is an effective visual strategy for tracking relatively fast targets, such as those in ball sports, since eye movement tracking with smooth pursuit can only handle relatively slow speeds. Therefore, the results of this study suggest that skilled ball sport players have acquired efficient head-tracking skills through VOR suppression. Previous studies focusing on hitters in fastball sports have shown that hitters tracked the ball by linking the rotation of their head to the motion of the ball, and that elite hitters directed their gaze to the ball using predictive saccades [30]. In contrast, because this study used a relatively simple catching task, it is likely that the skilled hitters stabilized their gaze rather than tracking the ball, which allowed them to make more efficient catching movements.

Previous studies have proposed two visual system hypotheses, which suggest that visual information used to perform actions (vision for action) and visual information relied upon when making perceptual judgments (vision for perception) are based on different neural circuits [31]. In this study, it was difficult to demonstrate distinctly whether the differences in eye movements between skilled and novice players were due to differences in their visual systems. However, from the results of this study, it can be inferred that either the skilled players were stabilizing their gaze and tracking the ball in their peripheral vision, or they were predicting the ball trajectory and did not need to track it entirely. Therefore, such gaze behavior in skilled players may at least reflect top–down behavior due to superior perception of visual information from previous experience and training, rather than bottom–up reflexive behavior.

## 5. Conclusions

This study attempted to determine visual strategies based on eye and head movements during a ball-catching task in a comparison of softball, tennis, and novice groups. Head and eye movement patterns during a ball-catching task were compared between subjects. The results showed that the softball group had a lower upward peak head velocity and downward peak eye velocity than the tennis and novice groups. In addition, when the head was pitched upward, a downward eye velocity was induced, indicating a VOR during head movement. Therefore, our results suggest that skilled ball players have relatively stable head and eye movements, which may lead to an effective gaze strategy. An advantage of the stationary gaze in the softball group could be to acquire visual information about the surroundings other than the ball. These results suggest that differences in ball-catching skills are not simply evaluated from the catching rate, but also from differences in visual strategy. Regarding the limitations of this study and future research, the direction of gaze, that is, where the player looks while catching the ball, is unknown since this study focused on the characteristics of eye and head movements in the ball catching task. By analyzing the details of gaze direction using an eye tracker system, it will be possible to clarify the characteristics of a more detailed gaze strategy, such as how the player looks at the ball during the catching task. Therefore, future research should examine not only eye and head movements, but also the relationship between the gaze and ball position in a comparison between skilled and novice players.

## Data Availability

The data supporting the conclusions of this article will be made available on request.
